# Strategies for Enhancing the Dielectric Constant of Organic Materials

**DOI:** 10.1021/acs.jpcc.2c05682

**Published:** 2022-10-10

**Authors:** Selim Sami, Riccardo Alessandri, Jeff B. W. Wijaya, Fabian Grünewald, Alex H. de Vries, Siewert J. Marrink, Ria Broer, Remco W. A. Havenith

**Affiliations:** † Stratingh Institute for Chemistry, University of Groningen, Nijenborgh 4, 9747 AGGroningen, The Netherlands; ‡ Zernike Institute for Advanced Materials, University of Groningen, Nijenborgh 4, 9747 AGGroningen, The Netherlands; § Groningen Biomolecular Sciences and Biotechnology Institute, University of Groningen, Nijenborgh 7, 9747 AGGroningen, The Netherlands; ∥ Department of Chemistry, Ghent University, Krijgslaan 281-(S3), B-9000Ghent, Belgium

## Abstract

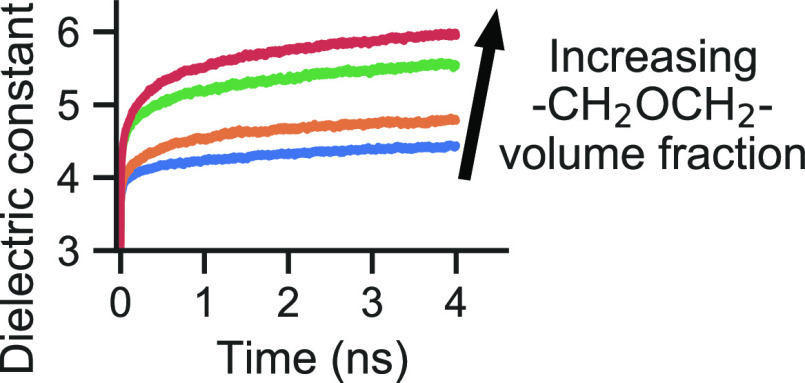

High dielectric constant organic semiconductors, often obtained by the use of ethylene glycol (EG) side chains, have gained attention in recent years in the efforts of improving the device performance for various applications. Dielectric constant enhancements due to EGs have been demonstrated extensively, but various effects, such as the choice of the particular molecule and the frequency and temperature regime, that determine the extent of this enhancement require further understanding. In this work, we study these effects by means of polarizable molecular dynamics simulations on a carefully selected set of fullerene derivatives with EG side chains. The selection allows studying the dielectric response in terms of both the number and length of EG chains and also the choice of the group connecting the fullerene to the EG chain. The computed time- and frequency-dependent dielectric responses reveal that the experimentally observed rise of the dielectric constant within the kilo/megahertz regime for some molecules is likely due to the highly stretched dielectric response of the EGs: the initial sharp increase over the first few nanoseconds is followed by a smaller but persistent increase in the range of microseconds. Additionally, our computational protocol allows the separation of different factors that contribute to the overall dielectric constant, providing insights to make several molecular design guides for future organic materials in order to enhance their dielectric constant further.

## Introduction

The virtually unlimited chemical space of organic molecules offers, in principle, the possibility of having a perfect molecule for every application, all the while making it harder to find this molecule. Finding design rules for specific applications helps navigate through this vast chemical space toward better performing devices with desired properties. The use of high dielectric constant materials is one such design rule that has attracted significant attention in the fields of organic photovoltaics (OPVs),^1−13^ organic thermoelectrics (OTEs),^14−20^ and organic transistors.^11,21−26^

A high dielectric constant weakens the Coulombic forces between separated charge carriers. A weaker attraction between the electron and the hole means lower recombination rates, which in turn has a positive impact on the charge separation and transport, and the overall device performance.^27−30^ Theoretical work from Koster et al.^31^ has shown that increasing the dielectric constant can result in an increased power conversion efficiency for OPVs and that with a sufficiently high dielectric constant (∼10), the excitonic behavior of OPVs can be avoided, with the condition that the energy offset required to enable charge transfer between the donor and the acceptor is minimized. However, even though recently, a dielectric constant above 10 has been obtained,^4^ the power conversion efficiency or the exciton binding energy of OPVs has not been shown yet to improve drastically. For OTEs, high dielectric constant organic materials have been shown to improve thermal stability, doping efficiency, and power factors.^14,15,17−19^ For organic transistors, it has been shown that strong Coulombic interactions have a detrimental effect on the charge carrier mobilities^32^ and that high dielectric constant organic materials can improve transport properties.^26^

The dielectric constant, despite what its name suggests, is not a constant: it is a property that is both frequency and temperature dependent. At high frequencies (∼terahertz), only the electrons are fast enough to respond to the electric field, giving the *electronic dielectric constant*. As the frequency decreases (gigahertz and below), the nuclear response, that is due to the reorientation of dipolar groups or entire molecules, can also contribute to the dielectric constant. In some materials, at even lower frequencies (kilohertz and below), the accumulation of space charges (i.e., mobile charges in particular regions of space such as interfaces or electrodes) can result in an additional increase in the dielectric constant. At the frequency where all of these contributions are fully active, the *static dielectric constant* can be obtained. Temperature change can hinder or unlock nuclear motion; therefore, it can have an important effect on the static dielectric constant, whereas the electronic dielectric constant is much less affected by it.

An important consequence of the frequency- and temperature-dependence of the dielectric constant is that for each organic electronic application, there exists a relevant frequency and temperature regime, and for each material, there exists an *effective dielectric constant* corresponding to those regimes. While the relevant temperature regime is trivially the operating temperature range of the device, the relevant frequency regime, that is, the slowest dielectric response that can still help the screening of charge carriers, is more elusive. We have previously established this threshold to be approximately in the gigahertz regime for OPVs and megahertz regime for the OTEs.^33^ Considering that most nuclear contributions are activated between these two regimes, the effective dielectric constant of a material for a specific application can correspond to either its static or electronic dielectric constant, or to a dielectric constant where the nuclear contributions are only partially activated. Since the design rules for maximizing the electronic (i.e., highly π-conjugated rigid backbone and high mass density) and the nuclear (i.e., highly polar and flexible side chains) dielectric contributions are contradictory,^33,34^ it is important to determine whether the nuclear dielectric contributions (partially or fully) improve the charge-carrier dynamics for a specific organic electronics application while designing new high *effective* dielectric constant materials.

**Figure 1:**
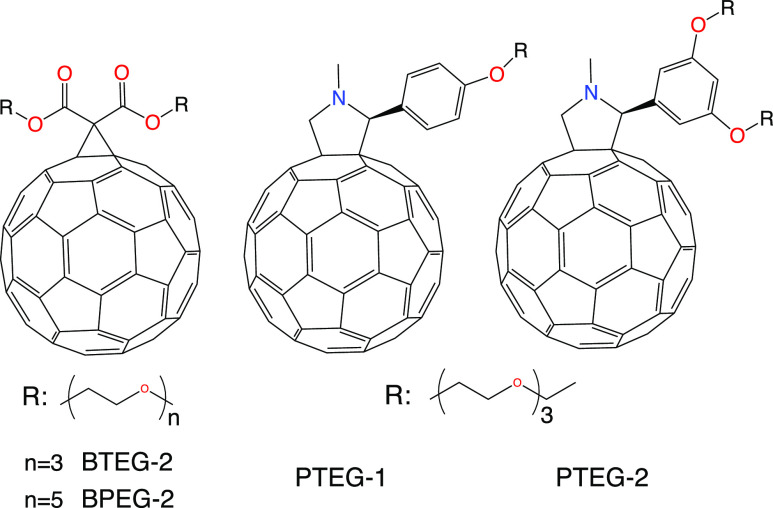
Molecules that are studied in this work.

A strategy that has been consistently successful and has become the preeminent way to obtain high *static* dielectric constant organic electronics is the use of ethylene glycol (EG) side chains.^30^ These side chains have been added to fullerene derivatives,^1−6,14−16,26^ small molecules,^7−9,35,36^ and polymers^10−13,17−25^ and have in turn resulted in increased static dielectric constants. Notably, recent work from Rousseva et al.^4^ reached a record static dielectric constant for fullerene derivatives (ϵ_r_ > 10) with the BPEG-2 molecule (see Figure 1). Rather unusually, the dielectric constant of these new fullerene derivatives shows a strong frequency dependence at low frequencies (below megahertz), which is hypothesized by the authors to be related to the higher flexibility of the EGs in these new molecules.^4^

While organic electronics have been widely studied through computational methods,^37−40^ computation of their dielectric properties has not been thoroughly explored. Electronic structure calculations, while accurate, are only computationally feasible for the computation of the electronic dielectric constant. On the other hand, classical fixed-charge molecular dynamics (MD) simulations are often not reliable for dielectric constant prediction.^41^ In a recent work,^33^ we have outlined a computational protocol that, using polarizable MD simulations, can calculate both the time- and frequency-dependent dielectric constant for organic solids. Moreover, we have identified the ability of EGs to partially align in response to the electric field, even in the constrained solid phase, as the mechanism that increases the dielectric constant. In this work, we apply this computational protocol to a carefully selected set of fullerene derivatives with EG side chains (see Figure 1) in order to understand their very different experimental dielectric constants.^4^ This selection of molecules allows comparing the effect of (1) having different number of EG chains (PTEG-1 vs PTEG-2); (2) having different length of EG chains (BTEG-2 vs BPEG-2); and (3) having the same number of EG units with different groups connecting the fullerene to the EG chain (PTEG-2 vs BTEG-2). Such a selection of molecules, combined with the molecular resolution of the simulations, allows the separation of different factors that contribute to the overall dielectric constant. Using these results, we are able to make several molecular design suggestions on how the dielectric constant can be further enhanced. Additionally, the computed frequency-dependent dielectric response reveals that the experimentally observed rise of the dielectric constant between the kilo/megahertz regime for some molecules is likely due to the highly stretched dielectric response of the EGs, where the initial sharp increase over the first few nanoseconds is followed by a small but persistent increase in the range of microseconds. Finally we show that while the dielectric constant decreases at lower temperatures, most of the nuclear contribution persists at temperatures that organic electronics can be expected to operate at.

## Results and Discussion

**Figure 2:**
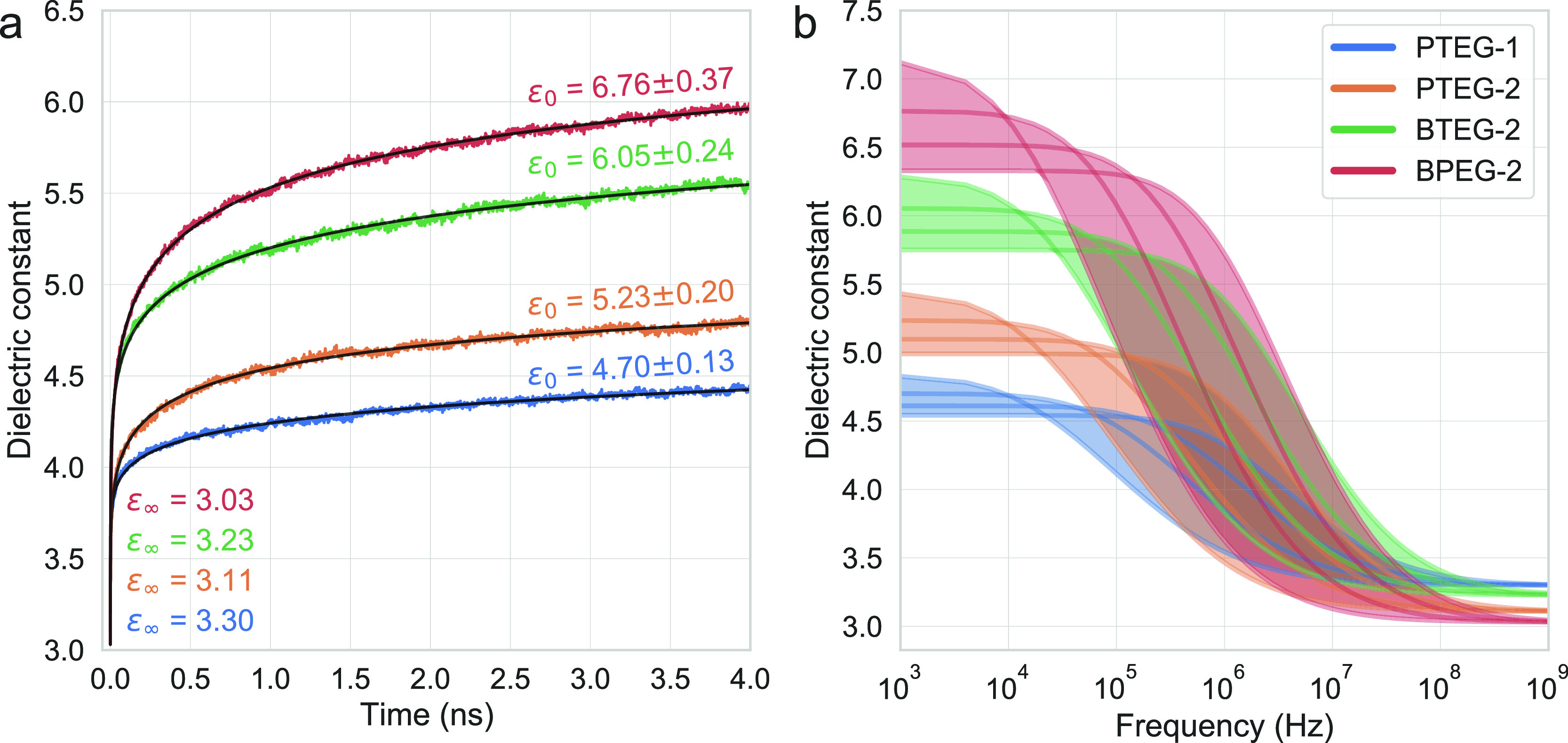
(a) Computed time- and (b) frequency-dependent dielectric constants for the PTEG-1, PTEG-2, BTEG-2, and BPEG-2 molecules at 298 K. Black lines in (a) correspond to the best fit with eq 2. ϵ_∞_ corresponds to the computed electronic dielectric constant, and ϵ_0_ corresponds to the extrapolated (with eq. 2) static dielectric constant. The estimated error margins of ϵ_0_ in (a), and the spread the dielectric constant over the frequencies in (b) correspond to using β values in eq 2, ranging from 0.22 to 0.28 as explained in the text. The bold lines in (b) for each molecule correspond to best fits with β values of 0.22, 0.24, 0.26, and 0.28 (higher β leads to higher transition frequency and lower ϵ_0_).

The computed time- and frequency-dependent dielectric constants of PTEG-1, PTEG-2, BTEG-2, and BPEG-2 (see Figure 1 for the structures) are shown in Figure 2. The results show that the electronic contribution (PTEG-1 > BTEG-2 > PTEG-2 > BPEG-2) decreases as the size of the side chain grows. This is due to the decrease in the volume fraction of the highly polarizable C_60_. The opposite is true when the nuclear contributions are added to the dielectric constant (BPEG-2 > BTEG-2 > PTEG-2 > PTEG-1) where the EG volume fraction seems to be the determining factor. These differences are further investigated by partitioning the dielectric response into molecular fragments. By fitting the dielectric response to a stretched exponential function (see the Methods section), the dielectric response time and the converged dielectric constant can be obtained. Additionally, by Fourier transforming that fit, the frequency-dependent dielectric constant can be obtained, as shown in Figure 2b. Due to the long extrapolation times, there is some level of uncertainty of the fit: we have seen that good fits to the dielectric response (Figure 2a, black lines) can be obtained for β = 0.22–0.28, where β is the stretching parameter of the exponential function (see eq 2 in the Methods section). Taking this uncertainty into consideration, static dielectric constants (ε_0_) of 4.70 ± 0.13, 5.23 ± 0.20, 6.05 ± 0.24, and 6.76 ± 0.37 are obtained for PTEG-1, PTEG-2, BTEG-2, and BPEG-2, respectively. Note that the computed static dielectric constant does not contain the contributions due to space charges as they are not present in the simulations. The dielectric response times corresponding to the different fits range between 0.3 and 1.3 ns, and the transition frequencies range between 0.1 and 5 MHz.

The computed dielectric constants are in good agreement with the experimental values for PTEG-1 (4.5–6.5)^1,42^ and PTEG-2 (5.4 ± 0.2),^1^ while they appear to be lower than the experimental values for BTEG-2 (7.5 ± 0.8)^4^ and BPEG-2 (9.8 ± 0.6),^4^ especially for BPEG-2. It is however difficult to determine the experimental nuclear response for BTEG-2 and BPEG-2 as there appears to be no plateau over the whole frequency regime, but instead, a linear-like (in logarithmic frequencies) increase. The authors suggest that this increase is due to the higher flexibility of EGs in these molecules. Our results indicate that this hypothesis is likely correct for the frequencies above 10^3^ Hz. However, as the dielectric response against the logarithmic frequency does not normally follow a linear increase, we suggest that this linear-like response is due to the overlap of two separate responses, dominated by the response of EGs above 10^3^ Hz and by space-charge responses below 10^3^ Hz. Our hypothesis that this linear-like response is a combination of two separate responses is strengthened when Figure 3 of Rousseva et al.,^4^ where the capacitance against the frequency is presented at 200 and 280 K, is carefully inspected: while the capacitance appears to be linearly increasing over the frequency range at 280 K for BTEG-2, at 200 K only a rise below 10^3^ Hz is observed, which we argue is due to space-charge effects and not EGs since their motion is seriously hindered at this temperature.

**Figure 3:**
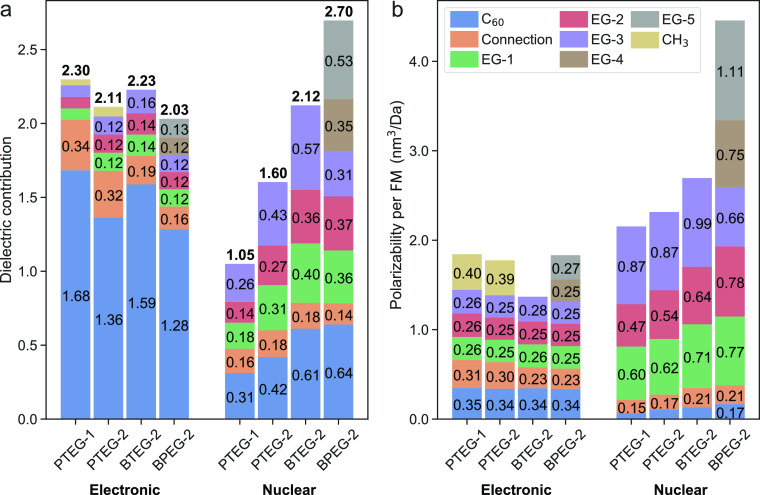
(a) Electronic and nuclear (at *t* = 4 ns) dielectric contributions of the different fragments of the studied molecules. Values in bold above the top of the bars correspond to the sum of all fragments. To obtain the dielectric constant, 1 (vacuum dielectric constant) must be added to the values in bold. (b) Values in (a) are multiplied by the volume of the simulation box in order to obtain the polarizability (dielectric contribution = polarizability/volume), then divided by the FM to obtain the polarizability per mass unit.

The dielectric contribution of these four molecules can be decomposed into molecular fragments and dielectric processes (Figure 3). While it is unusual to split the dielectric constant into fragment contributions, within the approximations of the performed polarizable MD simulations, this splitting is exact: polarization of every atom is known at every step, and using eq 1, the fragment contributions can be computed. In Figure 3a, contributions to the dielectric constant are shown, while in Figure 3b, polarizability per fragment mass (FM) is shown. The aim of the latter figure is to have a fair comparison of how much “work” (i.e., polarization per FM) each fragment in each molecule is doing. In both figures, the contributions are split into electronic and nuclear contributions at *t* = 4 ns. The EG molecular fragments correspond to *COC* repeating units in the side chain, which is terminated with an ethyl group (an additional CH_2_ unit) in the case of the PTEG series (PTEG-1 and PTEG-2) unlike the BXEG series (BTEG-2 and BPEG-2), which is terminated with a methyl group. The part between the C_60_ and the EG units corresponds to the *connection* fragment. In terms of the electronic dielectric constant, the results show that while C_60_ is responsible for most of the contribution, its polarizability per FM is rather comparable to other carbon-heavy fragments (CH_3_, connection of the PTEG series), suggesting that its high electronic dielectric constant is mostly because of its high mass density rather than its polarizability. The nuclear contributions, on the other hand, are clearly dominated by the EG units for all molecules. The nuclear C_60_ contribution increases as the EG volume fraction increases (BPEG-2 > BTEG-2 > PTEG-2 > PTEG-1), indicating that this contribution arises as a consequence of the EG dipole and C_60_-induced dipole interactions. The connection fragment for all molecules underperforms in terms of the dielectric constant, indicating the importance of minimizing the size of this unit, as was realized for the BXEG series.

Next, we discuss in detail the nuclear EG contributions in Figure 3 of the different molecules. Due to its higher flexibility, the terminal EG unit is shown to have the highest contribution for all molecules. Interestingly, for all molecules, the central EG chain (EG-3 for BPEG-2 and EG-2 for the rest) has the lowest contribution, suggesting that there is also some flexibility originating from the “connection” end of the side chain. EG units 1, 2, and 4 of BPEG-2 have comparable dielectric contributions, indicating that a longer side chain does not suffer from diminishing returns and that increasing the length of the side chain is a good strategy to maximize the dielectric constant. In fact, comparing the polarizability per FM of the EGs seems to indicate that the “work” (i.e., polarization per FM) each EG unit does increases as the volume fraction of EG units increases, likely due to the formation of larger EG domains that are more flexible. While still following this trend, PTEG-2 seems to somewhat underperform with respect to its number of EG chains.

**Figure 4:**
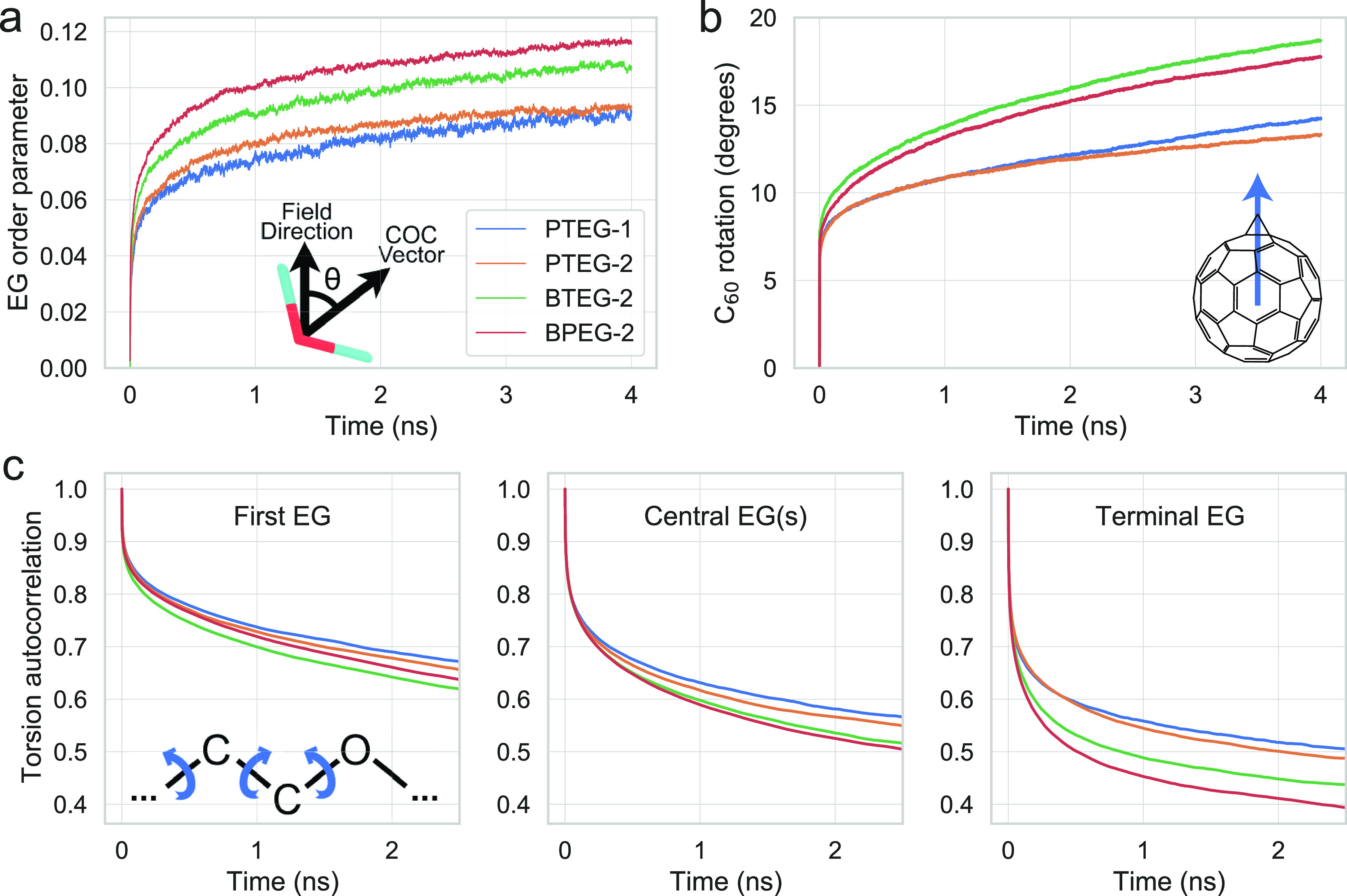
(a) Average P1 order parameter (⟨cos θ⟩) for the three EG units (see the inset figure) in PTEG-1, PTEG-2, and BTEG-2 and five EG units in BPEG-2; (b) angle between the C_60_ direction at *t* = 0 and the following times. The C_60_ direction is defined as the vector that points from the center of mass of C_60_ to the center of mass of the two connection carbons in C_60_ (see the inset figure); (c) autocorrelation function of the three torsions preceding the oxygen (see the inset figure) for the first, central (three central for BPEG-2), and terminal EGs. Autocorrelation is obtained from equilibrium simulations while EG order parameter and C_60_ rotation are from applied field simulations.

As shown in Figure 4a, the average order parameter P1 (⟨cos θ⟩) of the EG units is calculated to investigate the alignment of the EGs in different molecules in response to the applied electric field. Here, θ is the angle between the direction of the applied field and the *COC* vector (see inset figure). *P*1 = 0 corresponds to the random orientation of EGs, as it is at *t* = 0, which can be expected in an amorphous system. After the sudden application of an electric field, EGs are shown to align over time in the direction of the applied field in a similar exponentially decaying manner to the dielectric response that is shown in Figure 2. The relative alignment of each molecule (BPEG-2 > BTEG-2 > PTEG-2 ∼ PTEG-1) follows the same trend as the nuclear polarizability (Figure 3b) of the EG units of these molecules. This demonstrates that the order parameter is a good indication of the nuclear polarizability of each functional group, also considering the dipole moment when comparing different functional groups. Computation of this property with cheaper methods, such as non-polarizable MD, could be incorporated in a future workflow to approximate the dielectric response of different functional groups.

The alignment of EGs, as shown by the order parameter in Figure 4a, could occur as a consequence of different motions. The dominant contribution is expected to be the torsional flexibility of EG units, but small molecular tilts could also contribute to the overall alignment. To this end, we look at (1) the torsional flexibility of the first, central (three central ones averaged in the case of BPEG-2), and terminal EG units (Figure 4c) quantified by the average autocorrelation function of the three torsions preceding the oxygen and (2) the possibility of small molecular tilts quantified by the reorientation of the C_60_ moiety with respect to *t* = 0, as shown in Figure 4b. Note that this is not alignment but reorientation as C_60_ does not align with the electric field. It is shown in Figure 4c that the autocorrelation decays faster for the BXEG series than the PTEG series, indicating a higher flexibility for the former series. The largest difference between the two series in terms of the autocorrelation decay is seen for the terminal EG, which suggests that the additional CH_3_ group in the PTEG series negatively affects the flexibility and thus the dielectric contribution. The flexibility of the dihedrals is also shown to increase in all cases toward the end of the chain as fewer atoms need to take part in the rotation. Next, looking at the reorientation of the C_60_ moiety in Figure 4b, it can be seen that there is some amount of flexibility originating in the molecule from the C_60_. This is much higher for the BXEG series, indicating that a smaller “connection” group helps improve the rotational flexibility of the molecule. BPEG-2 has a slightly lower C_60_ flexibility than BTEG-2, which can be due to the longer side chain. While PTEG-1 and PTEG-2 have an almost identical C_60_ flexibility during the first 2 ns, PTEG-1 shows increased flexibility during the next 2 ns, indicating that the number of EG chains also has an influence on the C_60_ flexibility. Besides the number of EG chains, PTEG-1 and PTEG-2 also have different positioning of the EG chains on the benzene ring (para and meta), which might also play a role on the C_60_ flexibility.

**Figure 5:**
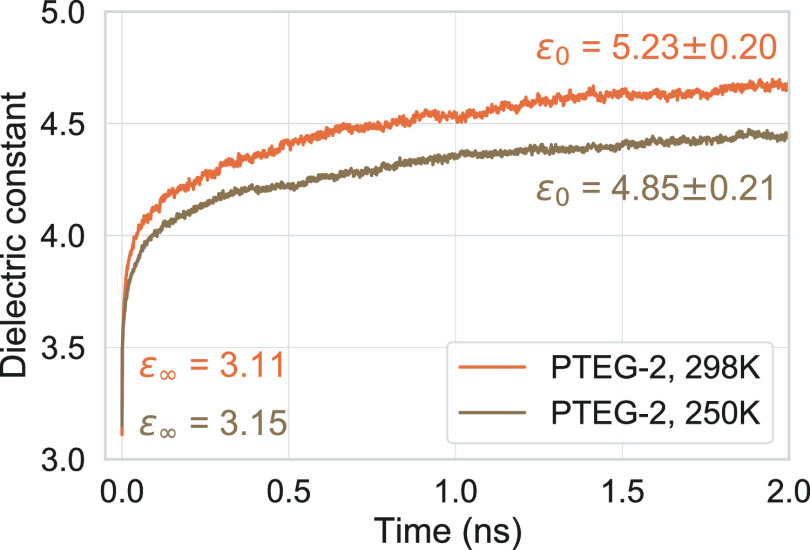
Temperature dependence of the time-dependent dielectric constant for PTEG-2, at 298 and 250 K. ϵ_∞_ corresponds to the computed electronic dielectric constant, and ϵ_0_ corresponds to the extrapolated (as explained in Figure 2 caption) static dielectric constant.

The dielectric constant is known to be a temperature-dependent property. In the case of PTEG-2, the temperature dependence of the capacitance at frequencies below the MHz regime has been investigated experimentally and has been shown to drop gradually as the temperature is lowered.^4^ In Figure 5, the computed dielectric responses of PTEG-2 at 298 and 250 K are shown. Similar to the experiments, a decrease in the dielectric response is observed at the lower temperature, indicating that the model can capture the temperature dependence of the dielectric response as well. A slightly higher electronic dielectric constant is discernible for 250 K (3.15) compared to 298 K (3.11), which is due to the higher mass density at the lower temperature. As soon as nuclei start responding to the electric field (*t* > 0), the dielectric constant at 298 K overtakes the one at 250 K due the higher available thermal energy enabling a larger range of nuclear motion. The difference between the two gradually increases over the simulation time, up to ∼0.25 at 2 ns, which corresponds to about a 15% decrease in the nuclear dielectric response. Based on the extrapolation method described earlier, we calculate the static dielectric constants to be 5.23 ± 0.20 and 4.85 ± 0.21 for 298 and 250 K, respectively. Considering that 250 K (−23.15 °C) is among the lowest temperatures that these devices could be expected to operate at, we can infer, at least for the PTEG-2 molecule, that the dielectric responses due to EGs are mostly active at the low-end of the temperature range of most organic electronic applications.

## Conclusions

We have studied the dielectric response of a carefully selected set of fullerene derivatives with EG side chains by performing and analyzing polarizable MD simulations. The set enables comparison of different numbers of EG chains (PTEG-1 vs PTEG-2) of different lengths of EG chains (BTEG-2 vs BPEG-2) and of having the same number of EG units but with different groups connecting the fullerene to the EG chain (PTEG-2 vs BTEG-2). In addition, our computational protocol allows for the separation of different factors that contribute to the overall dielectric constant, which enabled us to gain the following insights for the design of future high dielectric constant materials: (1) the first step is to decide whether a high electronic or high static dielectric constant is of interest as these have different molecular design requirements: while the former can be enhanced by π-conjugated and high mass density systems, the latter benefits from highly dipolar and flexible side chains; (2) longer EG chains do not suffer from diminishing returns and in fact we showed that the contribution per EG increases with longer EG chains; therefore, longer EG chains are a good strategy to further enhance the static dielectric constant; (3) the size of the group connecting the π-conjugated backbone (C_60_ in this case) to the flexible side chain (EGs in this case) should be minimized as its contribution to both electronic and nuclear dielectric constant is not favorable; (4) some flexibility of the side chain originates from small molecular tilts, and a small connection group has also been shown to improve the flexibility of the C_60_ fragment; and (5) terminating the EG side chain with a methyl rather than ethyl group improves both the flexibility and the nuclear dielectric response.

Additionally, the computed time- and frequency-dependent dielectric response revealed that the experimentally observed rise of the dielectric constant between the kilo/megahertz regime for the BXEG series^4^ is likely due to the highly stretched dielectric response of the EGs where the initial sharp increase over the first few nanoseconds is followed by a small but persistent increase in the range of microseconds. Based on these results, we suggest that the linear-like increase in the experimental dielectric constant below the MHz regime is due to the overlap of two separate responses that are dominated, respectively, by the response of EGs above 10^3^ Hz and by space-charge responses below 10^3^ Hz.

## Methods

### Computation of the Time- and Frequency-Dependent Dielectric Constant

The methodology for the computation of the time- and frequency-dependent dielectric constant was described in detail in previous work.^33^ Here, we briefly go over the main aspects of it. The external field method,^43−46^ where an electric field *E*_*i*_^ext^ is suddenly applied at *t* = 0 in the direction *i*, was used to compute the time-dependent dielectric constant ϵ_*i*_(*t*)
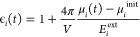
1where μ_*i*_(*t*) is the dipole moment, *V* is the volume of the simulation box, and μ_*i*_^init^ is the initial dipole moment before the electric field is applied. Then, in order to extrapolate the simulation to the converged response, ϵ(*t*) was fitted to a stretched exponential function^47,48^
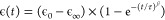
2where the three parameters are ϵ_0_, τ, and β, which are the static dielectric constant, the dielectric relaxation time, and the stretching parameter, respectively. Here, β = 1 corresponds to an ideal single-exponential dielectric response while 0 < β < 1 results in the stretching of the dielectric response due to a distribution of responses instead of a single one, as often observed in disordered solids.^47,49,50^ For the fullerene derivatives, β parameter varied from 0.22 to 0.28 in intervals of 0.02 instead of fitting, as fitting β resulted in instabilities in the fitting. ϵ_∞_ corresponds to the electronic dielectric constant, which was obtained using eq 1 at *t* = 0. Finally, the fit function was Fourier transformed in order to obtain the frequency-dependent dielectric constant.

### Polarizable MD Simulations

A Drude-based^51^ polarizable force field, where Drude particles’ positions are self-consistently relaxed at every MD step, was used for performing polarizable MD simulations. The force field was generated using the Q-Force methodology,^52,53^ which automatically derives force fields based on quantum mechanical calculations. Detailed information on the force field parametrization and validation can be found in the Supporting Information.

### Simulation and Analysis Protocol

The step-by-step simulation procedure for each molecule is as follows: (1) three amorphous morphologies with 125 molecules in the unit cell were generated using high-pressure simulations that are described in detail elsewhere;^33^ (2) for each morphology, 30 snapshots were taken with 100 ps intervals after an initial relaxation of 1 ns; (3) for each snapshot, three new simulations were started with an applied electric field in the *x*, *y*, or, *z* direction, which resulted in 280 simulations over which all of the results were averaged, resulting in a standard error of ∼0.02 for the dielectric constant for all systems; (4) the time-dependent dielectric constant was computed using eq 1 and was fitted with a stretched exponential using eq 2; and (5) the fitted function was Fourier transformed in order to obtain the frequency-dependent dielectric constant.

### MD Run Parameters

The double-precision version of GROMACS 2018.5^54^ software was used for all simulations. Leapfrog algorithm was used to integrate the equations of motion with a time step of 2 fs. The cutoff for non-bonded interactions was 1.4 nm. NPT ensemble was used with a temperature of 298 K and pressure of 1 bar, unless stated otherwise. The Berendsen^55^ thermostat (coupling parameter = 1 ps) and barostat (coupling parameter = 5 ps and compressibility = 4.5 × 10^–5^ bar^–1^) were used. The particle mesh Ewald method^56^ was used for the treatment of long-range electrostatic interactions. The strength of the applied electric field was 0.25711 V/nm (0.0005 au). The MD parameter file can be found in the Supporting Information.

## Supporting Information Available

Detailed information on the force field parametrization and validation (PDF)
Force field and coordinate files and scripts used for simulation and analysis (ZIP)
