# Supplementary material for: Strategies for Enhancing the Dielectric Constant of Organic Materials
Source: J Phys Chem C Nanomater Interfaces. 2022 Oct 10;126(45):19462–9. doi: 10.1021/acs.jpcc.2c05682 (PMC9677499; doi:10.1021/acs.jpcc.2c05682)
Supplement: Supplementary file 1 — jp2c05682_si_001.pdf [file jp2c05682_si_001.pdf]

Supporting Information: Strategies for Enhancing the Dielectric Constant of Organic Materials

Selim Sami,[*,†,‡,¶] Riccardo Alessandri,[‡,§,‖] Jeff B.W. Wijaya,[‡] Fabian Grünewald,[§] Alex H. de Vries,[§] Siewert J. Marrink,[‡,§] Ria Broer,[‡] and Remco W.A. Havenith[*,‡,†,⊥]

†*Stratingh Institute for Chemistry, University of Groningen, Nijenborgh 4, 9747 AG Groningen, The Netherlands*

‡*Zernike Institute for Advanced Materials, University of Groningen, Nijenborgh 4, 9747 AG Groningen, The Netherlands*

¶*Present address: Kenneth S. Pitzer Theory Center and Department of Chemistry, University of California, Berkeley, CA, USA*

§*Groningen Biomolecular Sciences and Biotechnology Institute, University of Groningen, Nijenborgh 7, 9747 AG Groningen, The Netherlands*

‖*Present address: Pritzker School of Molecular Engineering, University of Chicago, Chicago, IL 60637, USA*

⊥*Department of Chemistry, Ghent University, Krijgslaan 281-(S3), B-9000 Ghent, Belgium*

E-mail: s.sami@berkeley.edu; r.w.a.havenith@rug.nl

Contents

1 Parametrization and validation of the force fields **S-3**

1.1 Vibrational frequencies S-3

1.2 Torsional profile fitting S-4

1.3 CMAP protocol S-6

1.4 Non-bonded parameters S-8

1.5 Polarizability S-8

1.6 Test on small molecules S-9

References **S-10**

1 Parametrization and validation of the force fields

The Q-Force[S1,S2] toolkit was used for the parametrization of the molecules. The specific protocols used for obtaining the bonded and non-bonded terms for both the polarizable and non-polarizable force fields are described here in detail. Additionally, the treatment that was necessary for the BTEG-2 and BPEG-2 molecules, namely the CMAP protocol, is also explained here. All QM calculations were done in gas phase with density functional theory (PBE functional and 6-31+G* basis set). The QM calculations were performed with the Gaussian16[S3] software.

1.1 Vibrational frequencies

The correspondence of the QM and Q-Force vibrational frequencies are plotted in Figure S1 for PTEG-2, BTEG-2, and BPEG-2 together. As can be seen, a good match between the two is obtained for all molecules with mean absolute errors of 2.83%, 3.14%, 3.34% for PTEG-2, BTEG-2, and BPEG-2, respectively.

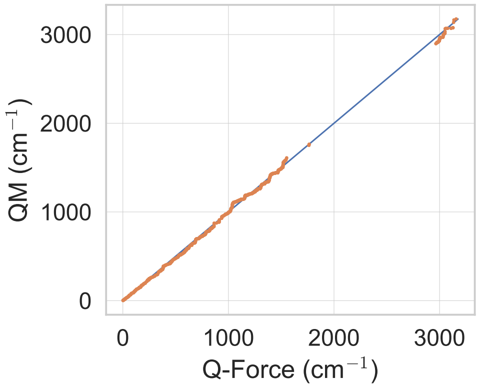

Figure S1: QM vs Q-Force vibrational frequencies (orange dots) for PTEG-1, PTEG-2, BTEG-2, and BPEG-2. The blue line corresponds to the perfect agreement between the two methods. Mean absolute errors of 2.74%, 2.83%, 3.14%, 3.34% are obtained for PTEG-1, PTEG-2, BTEG-2, and BPEG-2, respectively.

1.2 Torsional profile fitting

For all of the flexible torsions in the molecules, both QM and MM torsional scans are performed in order to match the two profiles, as previously described in detail.[S1] The results of this approach are shown in Figure S2 for the BTEG-2 molecule where a good match is obtained between the QM and MD profiles in all cases. BPEG-2 has almost identical profiles (with a longer chain) to BTEG-2. Similar results are shown in S3 for PTEG-1, which is almost identical (with two chains) for PTEG-2. The dihedral number 1 shown in the side chain representation in S2 is not treated at this step and will be discussed and validated in the next subsection.

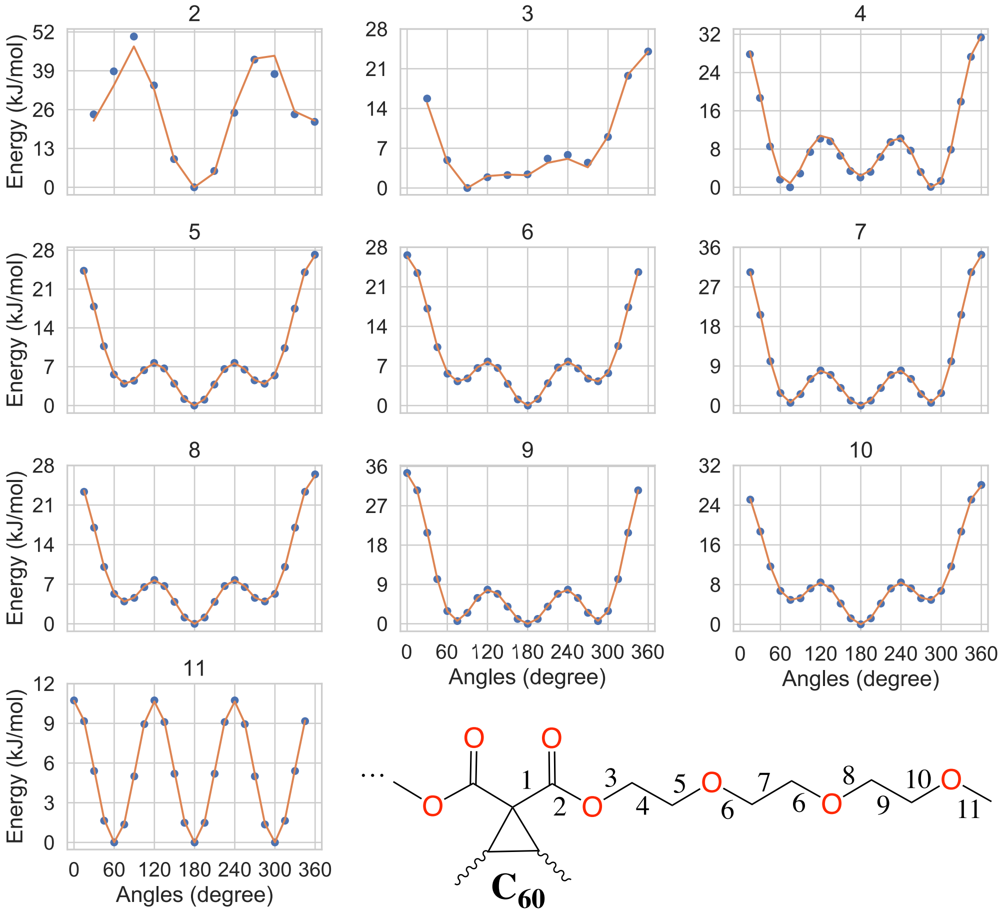

Figure S2: QM (blue dots) vs fitted Q-Force (orange lines) torsional energy profiles for the flexible torsions of BTEG-2. The number above each plot corresponds to the torsion numbers shown on the side chain representation below. Validation of torsion 1 is done at the CMAP subsection.

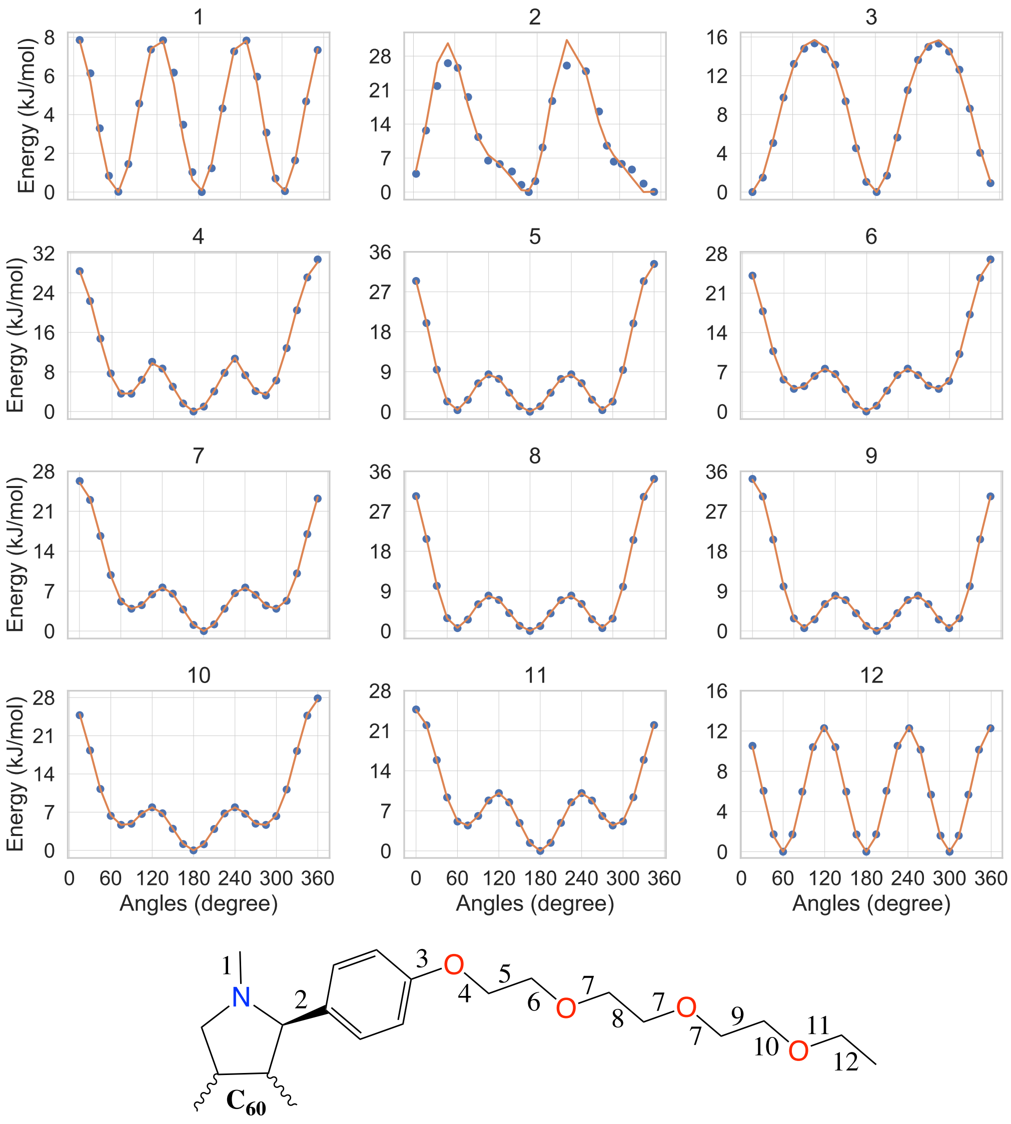

Figure S3: QM (blue dots) vs fitted Q-Force (orange lines) torsional energy profiles for the flexible torsions of PTEG-1. The number above each plot corresponds to the torsion numbers shown on the side chain representation below.

1.3 CMAP protocol

In some cases, two neighboring torsions can be mutually dependent, meaning that the minimum of one torsion can depend on the current position of the other torsion. While in some simple cases this dependence is directly captured by the non-bonded interactions so that no further action is necessary, some more complicated profiles require a two dimensional torsional correction map, often called a CMAP.[S4–S6] Such a CMAP was necessary for the BTEG-2 and BPEG-2 molecules for the torsion pair shown in Figure S4. To determine the correction map, these two dihedrals were scanned both with QM and MM for a BTEG-2 type fullerene derivative with minimal side chains, as shown in Figure S4. Then, the difference between the two profiles was used as the correction map. Note that in this approach, the non-bonded interactions of the side chain (except the terminal carbons) with itself and with the C_{60} were turned off. The good agreement between the 2D QM (left) and the corrected MM profile (right) is shown in Figure S4. The mutual dependence of the two torsions can be seen in the profiles; i) there are four global minima regions (light green), which correspond to one torsion being perpendicular and one parallel to the C_{60} and the switch between the pairs of these minima corresponds to a very small energy barrrier (~4 kJ/mol). A full 360° rotation of the torsions is also possible through a concerted movement of both, which has an energy barrier of ~12 kJ/mol (dark green).

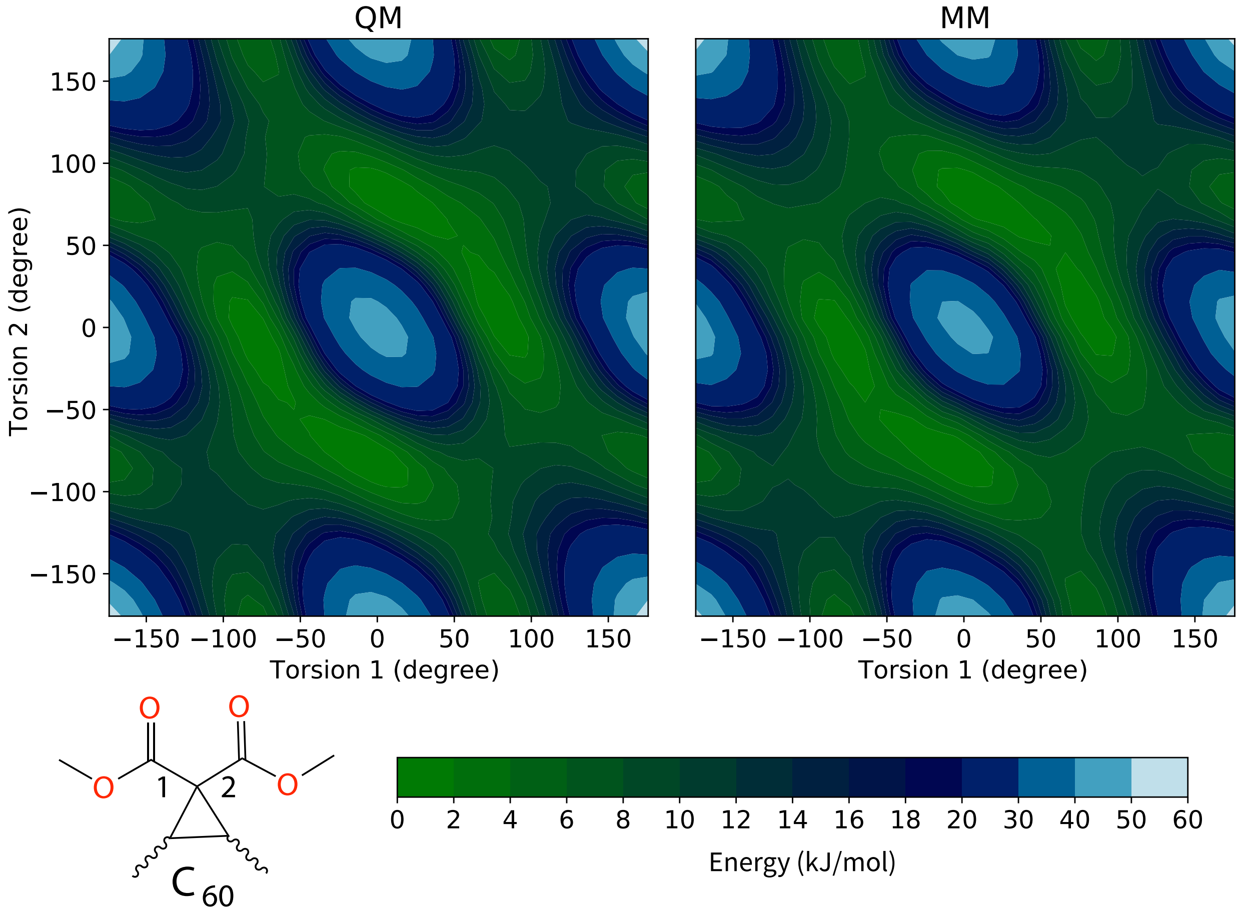

Figure S4: 2D torsional profiles (left: QM, right: MM) for the two torsions depicted in the inset figure. The correlation between the two torsions can be understood from the fact that the energy landscape of one torsion strongly depends on the current position of the other.

1.4 Non-bonded parameters

Point charges were calculated using the Hirshfeld partitioning[S7] based CM5 method.[S8] For the Lennard-Jones parameters, the default GROMOS 54a6 parameters were used for both the polarizable and the non-polarizable versions of the force field. In case of the polarizable force field, dispersion interactions (C_6 parameters) were scaled down by 20% in order to prevent double-counting as C_6 parameters in non-polarizable force fields are often enhanced to partially account for polarizability.[S9] It is also shown in a later subsection that such an approach give thermodynamic properties that are in good agreement with the experimental ones for small test molecules. Lennard-Jones parameters for the C_{60} carbons were obtained from the work of Girifalco.[S10,S11]

1.5 Polarizability

Drude particles[S12] were added to all atoms for the inclusion of polarizability in the simulations. Before each MD step, the positions of these particles were minimized iteratively to polarize the system. We found that the use of the united atom polarizabilities from Visscher et al.[S9] provided consistently low electronic dielectric constants, therefore, these parameters were scaled up by 20% in order to obtain electronic dielectric constants that are comparable to the experimental ones, as it will be shown in the next subsection. Polarizabilities for the C_{60} carbons were obtained from experimental results[S13] which resulted in an electronic dielectric constant (4.05) for crystalline C_{60} that is in good agreement with the same experimental work (4.08±0.05). The combination of these two different sources for the atomic polarizabilities, together with the same Q-Force procedure for the remainder of the force field, resulted for amorphous PCBM to an electronic dielectric constant of 3.39, in excellent agreement with experimental work (3.4).[S14]

1.6 Test on small molecules

In order to determine the validity of the parameterization scheme before moving on to the fullerene derivatives, polarizable force fields derived with the exact same methodology described above were tested on two EG-based molecules diglyme (CH_3O–[–CH_2CH_2O–]$_2$–CH_3) and tetraglyme (CH_3O–[–CH_2CH_2O–]$_4$–CH_3). As shown in Table S1, both the thermodynamic and the dielectric properties for both molecules are in good agreement with the experimental references. Notably, the dielectric relaxation times (τ) for liquid EG bearing molecules are much faster than PTEG-1, due to easier molecular reorientation. Similarly, much higher stretching coefficients (β) are obtained for these liquid systems, meaning that the dielectric response is much closer to an ideal single-exponential dielectric response.

Table S1: Thermodynamic and dielectric properties of diglyme (DG) and tetraglyme (TG). ρ = density (g/cm^3), ΔH_{vap} = enthalpy of vaporization (kJ/mol), ϵ_∞ = electronic dielectric constant, ϵ_0 = static dielectric constant, τ = dielectric relaxation time (ps), β = stretching coefficient of the exponential fit from eq. 2 of main text, ω_{max} = transition frequency of the dielectric constant (GHz).

Molecules	ρ	ΔH_{vap}	ϵ_∞	ϵ_0	τ	β	ω_{max}
DG (calc.)	895.3	49.7	1.94	7.6	9.4	0.77	10
DG (exp.)	940±2[S15]	48.0±0.6[S16]	1.99[S17]	7.3, 7.4[S15]	6.9-11.8,[S18]	-	-
TG(calc.)	970.4	80	2.01	7.8	19.5	0.69	4
TG(exp.)	1006±1[S15]	76.9±2.6[S16]	2.05[S15]	7.78[S15]	12-18.2[S18]	-	-

References

(S1) Sami, S.; Menger, M. F.; Faraji, S.; Broer, R.; Havenith, R. W. A. Q-Force: Quantum Mechanically Augmented Molecular Force Fields. *J. Chem. Theory Comput.* **2021**, *17*, 4946–4960.

(S2) Sami, S.; Menger, M. F. Q-Force: Quantum mechanically augmented molecular force fields. `https://github.com/selimsami/qforce`, commit ID: 8bc6277 (accessed July 2020).

(S3) Frisch, M. J.; Trucks, G. W.; Schlegel, H. B.; Scuseria, G. E.; Robb, M. A.; Cheeseman, J. R.; Scalmani, G.; Barone, V.; Petersson, G. A.; Nakatsuji, H.; Li, X.; Caricato, M.; Marenich, A. V.; Bloino, J.; Janesko, B. G.; Gomperts, R.; Mennucci, B.; Hratchian, H. P.; Ortiz, J. V.; Izmaylov, A. F.; Sonnenberg, J. L.; Williams-Young, D.; Ding, F.; Lipparini, F.; Egidi, F.; Goings, J.; Peng, B.; Petrone, A.; Henderson, T.; Ranasinghe, D.; Zakrzewski, V. G.; Gao, J.; Rega, N.; Zheng, G.; Liang, W.; Hada, M.; Ehara, M.; Toyota, K.; Fukuda, R.; Hasegawa, J.; Ishida, M.; Nakajima, T.; Honda, Y.; Kitao, O.; Nakai, H.; Vreven, T.; Throssell, K.; Montgomery, J. A., Jr.; Peralta, J. E.; Ogliaro, F.; Bearpark, M. J.; Heyd, J. J.; Brothers, E. N.; Kudin, K. N.; Staroverov, V. N.; Keith, T. A.; Kobayashi, R.; Normand, J.; Raghavachari, K.; Rendell, A. P.; Burant, J. C.; Iyengar, S. S.; Tomasi, J.; Cossi, M.; Millam, J. M.; Klene, M.; Adamo, C.; Cammi, R.; Ochterski, J. W.; Martin, R. L.; Morokuma, K.; Farkas, O.; Foresman, J. B.; Fox, D. J. Gaussian 16. `https://gaussian.com`, 2016; Gaussian Inc. Wallingford CT.

(S4) MacKerell, A. D.; Feig, M.; Brooks, C. L. Improved treatment of the protein backbone in empirical force fields. *J. Am. Chem. Soc.* **2004**, *126*, 698–699.

(S5) Best, R. B.; Zhu, X.; Shim, J.; Lopes, P. E. M.; Mittal, J.; Feig, M.; MacKerell, A. D. Optimization of the additive CHARMM all-atom protein force field targeting improved

sampling of the backbone φ, ψ and side-chain χ_1 and χ_2 dihedral angles. *J. Chem. Theory Comput.* **2012**, *8*, 3257–3273.

(S6) Mackerell Jr., A. D. Empirical force fields for biological macromolecules: Overview and issues. *J. Comput. Chem.* **2004**, *25*, 1584–1604.

(S7) Hirshfeld, F. L. Bonded-atom fragments for describing molecular charge densities. *Theoret. Chim. Acta* **1977**, *44*, 129–138.

(S8) Marenich, A. V.; Jerome, S. V.; Cramer, C. J.; Truhlar, D. G. Charge model 5: An extension of Hirshfeld population analysis for the accurate description of molecular interactions in gaseous and condensed phases. *J. Chem. Theory Comput.* **2012**, *8*, 527–541.

(S9) Visscher, K. M.; Geerke, D. P. Deriving a Polarizable Force Field for Biomolecular Building Blocks with Minimal Empirical Calibration. *J. Phys. Chem. B* **2020**, *124*, 1628–1636.

(S10) Girifalco, L. A. Interaction potential for carbon (C60) molecules. *J. Phys. Chem.* **1991**, *95*, 5370–5371.

(S11) Girifalco, L. A. Molecular properties of fullerene in the gas and solid phases. *J. Phys. Chem.* **1992**, *96*, 858–861.

(S12) Lemkul, J. A.; Huang, J.; Roux, B.; MacKerell, A. D. An Empirical Polarizable Force Field Based on the Classical Drude Oscillator Model: Development History and Recent Applications. *Chem. Rev.* **2016**, *116*, 4983–5013.

(S13) Eklund, P. C.; Rao, A. M.; Wang, Y.; Zhou, P.; Wang, K. A.; Holden, J. M.; Dresselhaus, M. S.; Dresselhaus, G. Optical-Properties of C_{60}-Based and C_{70}-Based Solid Films. *Thin Solid Films* **1995**, *257*, 211–232.

(S14) Guilbert, A. A. Y.; Schmidt, M.; Bruno, A.; Yao, J.; King, S.; Tuladhar, S. M.; Kirchartz, T.; Alonso, M. I.; Goñi, A. R.; Stingelin, N.; Haque, S. A.; Campoy-Quiles, M.; Nelson, J. Spectroscopic Evaluation of Mixing and Crystallinity of Fullerenes in Bulk Heterojunctions. *Adv. Funct. Mater.* **2014**, *24*, 6972–6980.

(S15) Riadigos, C.; Iglesias, R.; Rivas, M.; Iglesias, T. Permittivity and density of the systems (monoglyme, diglyme, triglyme, or tetraglyme+n-heptane) at several temperatures. *J Chem. Thermodynamics* **2011**, *43*, 275 – 283.

(S16) Nichols, G.; Orf, J.; Reiter, S. M.; Chickos, J.; Gokel, G. W. The vaporization enthalpies of some crown and polyethers by correlation gas chromatography. *Thermochim. Acta* **2000**, *346*, 15 – 28.

(S17) Lide, D.; Milne, G. *Handbook of Data on Organic Compounds*; CRC Press, Inc. Boca Raton, Florida, 1994; Vol. 1; p 2648.

(S18) Kowert, B. A.; Thurman-Keup, E. M.; Stemmler, A. J.; Stemmler, T. L.; Fehr, M. J.; Caldwell, C. V. C.; Gentemann, S. J. Electron Spin Resonance Studies of the Reorientational Motion of $Ni(mnt)_2^-$. *J. Phys. Chem. B* **2010**, *114*, 2760–2765.
